# Comparative physiological, metabolomic, and transcriptomic analyses reveal mechanisms of improved abiotic stress resistance in bermudagrass [*Cynodon dactylon* (L). Pers.] by exogenous melatonin

**DOI:** 10.1093/jxb/eru373

**Published:** 2014-09-15

**Authors:** Haitao Shi, Chuan Jiang, Tiantian Ye, Dun-xian Tan, Russel J. Reiter, Heng Zhang, Renyi Liu, Zhulong Chan

**Affiliations:** ^1^Key Laboratory of Plant Germplasm Enhancement and Specialty Agriculture, Wuhan Botanical Garden, Chinese Academy of Sciences, Wuhan, 430074, China; ^2^Shanghai Center for Plant Stress Biology, Shanghai Institutes for Biological Sciences, Chinese Academy of Sciences, Shanghai, 201602, China; ^3^University of Chinese Academy of Sciences, Beijing, 100039, China; ^4^Department of Cellular and Structural Biology, The University of Texas Health Science Center, San Antonio, TX, USA

**Keywords:** Abiotic stress, antioxidant, bermudagrass, melatonin, metabolites, reactive oxygen species, transcriptomic.

## Abstract

Melatonin (*N*-acetyl-5-methoxytryptamine), a well-known animal hormone, is also involved in plant development and abiotic stress responses. In this study, it is shown that exogenous application of melatonin conferred improved salt, drought, and cold stress resistances in bermudagrass. Moreover, exogenous melatonin treatment alleviated reactive oxygen species (ROS) burst and cell damage induced by abiotic stress; this involved activation of several antioxidants. Additionally, melatonin-pre-treated plants exhibited higher concentrations of 54 metabolites, including amino acids, organic acids, sugars, and sugar alcohols, than non-treated plants under abiotic stress conditions. Genome-wide transcriptomic profiling identified 3933 transcripts (2361 up-regulated and 1572 down-regulated) that were differentially expressed in melatonin-treated plants versus controls. Pathway and gene ontology (GO) term enrichment analyses revealed that genes involved in nitrogen metabolism, major carbohydrate metabolism, tricarboxylic acid (TCA)/org transformation, transport, hormone metabolism, metal handling, redox, and secondary metabolism were over-represented after melatonin pre-treatment. Taken together, this study provides the first evidence of the protective roles of exogenous melatonin in the bermudagrass response to abiotic stresses, partially via activation of antioxidants and modulation of metabolic homeostasis. Notably, metabolic and transcriptomic analyses showed that the underlying mechanisms of melatonin could involve major reorientation of photorespiratory and carbohydrate and nitrogen metabolism.

## Summary

Exogenous melatonin application confers abiotic stress resistance in bermudagrass through modulation of antioxidants and metabolic homeostasis, and extensive transcriptional reprogramming such as the reorientation of photorespiratory, carbohydrate, and nitrogen metabolism.

## Introduction

Melatonin (*N*-acetyl-5-methoxytryptamine) is a well-known animal hormone that is involved in many biological processes including sleep, mood, circadian rhythms, retinal physiology, seasonal reproductive physiology, temperature homeostasis, sexual behaviour, antioxidative activity, and immunological enhancement (Galano *et al.*, 2011; Venegas *et al.*, 2012; Calvo *et al.*, 2013). However, melatonin is not only found exclusively in animals, but is ubiquitously present in almost all forms of life including protists, prokaryotes, eukaryotic unicells, algae, fungi, and plants (Dubbels *et al.*, 1995; Hattori *et al.*, 1995; Kolář and Macháčkova, 2005; Arnao and Hernández-Ruiz, 2006, 2007; Tan *et al*., 2007*a*, *b*, 2012).

In 1995, two reports first identified melatonin in higher plants (Dubbels *et al.*, 1995; Hattori *et al.*, 1995). In the last 20 years, additional research found that melatonin is universally distributed in leaves, roots, stems, fruits, and seeds of all plant species examined (Manchester *et al.*, 2000; Reiter *et al.*, 2001; Kolář and Macháčkova, 2005; Hernández-Ruiz and Arnao, 2008; Murch *et al.*, 2009; Zhao *et al.*, 2013). Interestingly, remarkably high concentrations of melatonin have been identified and quantified in popular beverages (beer, tea, coffee, and wine), crops (barley, corn, grape, wheat, rice, tobacco, and oats), and *Arabidopsis* in comparison with those in animals (Manchester *et al.*, 2000; Kolář and Macháčkova, 2005; Arnao and Hernández-Ruiz, 2006, 2009*b*, 2013*a*, b; Tan *et al.*, 2007*a*, 2012; Ramakrishna *et al.*, 2012). Additionally, the melatonin content of tomato and rice has been modified by genetic engineering (Okazaki and Ezura, 2009; Okazaki *et al.*, 2009, 2010; Byeon *et al*., 2012, 2013, 2014; Byeon and Back, 2014*a*, b). The well-known beneficial effects of melatonin on human health and the abundance of melatonin in popular beverages and crops may encourage the daily consumption of these products (Tan *et al.*, 2012).

To date, melatonin has also been found to be a ubiquitous modulator in multiple plant developmental processes and various stress responses (Kolář and Macháčkova, 2005; Arnao and Hernández-Ruiz, 2006; Tan *et al.*, 2007*a*, 2012). Changes in melatonin in plants may be involved in circadian rhythms, flowering, promotion of photosynthesis, preservation of chlorophyll (Arnao and Hernández-Ruiz, 2009*a*; Tan *et al.*, 2012), stimulation and regeneration of root system architecture (Hernández-Ruiz *et al.*, 2005; Pelagio-Flores *et al.*, 2012; Zhang *et al.*, 2014), delayed senescence of leaves (Byeon *et al.*, 2012; Wang *et al*., 2012, 2013 *a*, *b*), and alleviation of oxidative damage mediated by reactive oxygen species (ROS) and reactive nitrogen species (RNS) burst (Tan *et al.*, 2012) Moreover, melatonin protects against multiple abiotic processes such as cold stress (Posmyk *et al.*, 2009*a*; Kang *et al.*, 2010; Bajwa *et al.*, 2014), copper stress (Posmyk *et al.*, 2008, 2009b), high temperature (Byeon and Back 2014*b*), salt stress (Li *et al.*, 2012), osmotic stress (Zhang *et al.*, 2013), drought stress (Wang *et al.*, 2014), and pathogen infection (Yin *et al.*, 2013). The mechanisms were partially characterized after the direct exogenous application of melatonin to plants (Posmyk *et al.*, 2008, 2009a, b; Zhao *et al.*, 2011; Li *et al.*, 2012; Pelagio-Flores *et al.*, 2012; Wang *et al*., 2012, 2013*a*, b; Yin *et al.*, 2013; Bajwa *et al.*, 2014) or the creation of transgenic plants that produced either more or less melatonin through modulating its metabolic pathway (Kang *et al.*, 2010; Byeon *et al.*, 2013, 2014; Park *et al.*, 2013; Byeon and Back, 2014*a*; Wang *et al.*, 2014). Finally, the recent studies which showed the protective roles of melatonin in response to abiotic stress indicate that this indole might be a potentially ideal target for future genetic engineering technology to improve abiotic stress resistance in plants. Thus, transgenic plants with higher melatonin concentration might lead to breakthroughs to improve crop production in agriculture as well as the general health of humans (Tan *et al.*, 2012).

Bermudagrass [*Cynodon dactylon* (L). Pers.] is a warm-season turfgrass for lawns, parks, and sport fields cultivated worldwide (Shi *et al*., 2012, 2013a, *b*, 2014 b, c, d). In response to global changed environmental stresses, improvement of abiotic stress resistance is very important for grass engineering (Shi *et al*., 2012, 2013a, *b*, 2014 b, c, d). As mentioned above, melatonin might be an ideal target for future genetic engineering of some plant species. However, the endogenous melatonin concentration and the possible role of melatonin in response to abiotic stress in bermudagrass is largely unknown. In this study, endogenous melatonin was examined after abiotic stress treatments in bermudagrass plants, and exogenous melatonin treatment was applied to investigate the *in vivo* role of melatonin in the response of bermudagrass to abiotic stress. In addition, the effects of exogenous melatonin treatment on ROS accumulation and cell damage, as well as underlying antioxidant responses, were determined. Moreover, comparative metabolomic and transcriptomic analyses were performed to identify differentially expressed metabolites and genes after exogenous melatonin treatment. This study provided some insights into the physiological and molecular mechanisms of melatonin in bermudagrass responses to multiple abiotic stresses.

## Materials and methods

### Plant materials and growth conditions

Newly harvested common bermudagrass seeds were used in this study. After stratification in deionized water at 4 °C for 4 d in darkness, the bermudagrass seeds were sown in soil in the growth room, which was controlled at 28 °C, with an irradiance of ~150 μmol quanta m^–2^ s^–1^, 16h light and 8h dark cycles, and ~65% relative humidity.

### Plant abiotic stress treatment

To test the effect of exogenous melatonin on plant physiological responses and abiotic stress resistance, 21-day-old bermudagrass plants were irrigated with water or with different concentrations of melatonin solutions for 7 d, respectively. After melatonin pre-treatment, all control and melatonin-pre-treated 28-day-old bermudagrass plants were subjected to subsequent salt, drought, or cold stress treatments. For salt stress treatment, 28-day-old bermudagrass plants were irrigated with NaCl solutions for 25 d; the NaCl concentration was increased stepwise by 50mM every 5 d to a final concentration of 300mM. For drought stress treatment, 28-day-old plants were subjected to a drought condition by withholding water for 21 d and then re-watered for 4 d. For cold stress treatment, 28-day-old bermudagrass plants were subjected to 4 °C treatment for 21 d, and then transferred to –10 °C for 8h. The freezing stress-treated plants were then recovered overnight at 4 °C and transferred to a standard growth room (28 °C) for 4 d. In each independent experiment, three pots with ~40 plants in each pot were used for each treatment in one concentration of melatonin, and at least three independent experiments were performed to obtain the results.

The survival rate of the salt-, drought-, or freezing-stressed plants was recorded at 25 d after stress treatments. The plant leaf samples from melatonin-pre-treated 28-day-old plants were collected at the indicated time points after salt, drought, or cold treatment for the assays of multiple of physiological parameters.

### Quantification of melatonin by enzyme-linked immunosorbent assay (ELISA)

Melatonin from plant leaves was extracted using the acetone–methanol method as described by Pape and Lüning (2006). Briefly, 1g of plant leaf samples was ground in liquid nitrogen, and then transferred to 5ml of extraction mixture (acetone:methanol:water=89:10:1) and homogenized extensively on ice, and the homogenate was centrifuged at 4500 *g* for 5min at 4 °C. The supernatant was moved to a new centrifuge tube containing 0.5ml of 1% trichloric acid for protein precipitation. After centrifugation at 12 000 *g* for 10min at 4 °C, the extract was used for quantification of melatonin using the Melatonin ELISA Kit (EK-DSM; Buhlmann Laboratories AG, Schonenbuch, Switzerland) according to the manufacturer’s instruction as described in Shi and Chan (2014*a*).

### Quantifications of chlorophyll content

Plant leaf chlorophyll was extracted using 80% (v/v) acetone for 6h with shaking in the dark. The concentration of chlorophyll was then calculated by examining the absorbance at 645nm and 663nm of the centrifuged supernatant.

### Quantification of electrolyte leakage (EL)

The EL of plant leaves under control and abiotic stress conditions was assayed using a conductivity meter (Leici-DDS-307A, Shanghai, China) as previously described (Shi *et al*., 2012, 2013a, *b*, 2014 b, c, d). The relative EL was expressed as the ratio of initial conductivity to the conductivity after boiling.

### Determination of malondialdehyde (MDA) content

The MDA content was extracted using chilled thiobarbituric acid (TBA) reagent, and was quantified via determining the absorbance of the supernatant at 450, 532, and 600nm as previously described (Shi *et al*., 2012, 2013a, *b*, 2014 b, d).

### Determination of ROS accumulation and antioxidants

As two major indicators of ROS accumulation, hydrogen peroxide (H_2_O_2_) and superoxide radical (O_2_·^–^) contents were quantified using the titanium sulphate method and the Plant O_2_·^–^ ELISA Kit (10-40-488, Bejing Dingguo, Beijing, China) as previously described (Shi *et al*., 2012, 2013*a*, *b*, 2014 *b*, *d*).

The activities of three antioxidant enzymes, namely superoxide dismutase (SOD; EC 1.15.1.1), catalase (CAT; EC 1.11.1.6). and peroxidase (POD; EC 1.11.1.7), were assayed using a Total SOD Assay Kit (S0102; Haimen Beyotime, Haimen city, China), a CAT Assay Kit (S0051; Haimen Beyotime), and a Plant POD Assay Kit (A084-3; Nanjing Jiancheng, Nanjing city, China), respectively, as described by Shi *et al*., 2012, 2013a, *b*, 2014 b, d). The concentrations of reduced glutathione (GSH) and oxidized glutathione (GSSG) were determined using the GSH and GSSG Assay Kit (S0053; Haimen Beyotime) as described by Shi *et al*. (2014*b*, *d*), and the GSH redox state was calculated as the ratio of GSH concentration to the concentration of GSH plus GSSG.

### Extraction, identification, and quantification of metabolites

Extraction, identification, and quantification of metabolites from plant leaves were performed as in Shi *et al.* (2014*d*). Briefly, the metabolite extraction and sample derivatization were performed as in Lisec *et al.* (2006), then the derivatizated extract was injected into a DB-5MS capillary cloumn (30 m×0.25 mm×0.25 μm; Agilent J&W GC column, California, USA) using gas chromatography time-of-flight mass spectrometry (GC-TOF-MS) (Agilent 7890A/5975C) according to the protocol described by Shi *et al.* (2014*d*). After the GC-TOF-MS assay, the various metabolites were identified via comparing every retention time index-specific mass with reference spectra in mass spectral libraries (NIST 2005, Wiley 7.0). The numerous metabolites were then quantified based on the pre-added internal standard (ribitol) in the process of metabolite extraction.

### Hierarchical cluster analysis

The hierarchical cluster analysis of several metabolites was performed using the CLUSTER program (http://bonsai.ims.u-tokyo.ac.jp/~mdehoon/software/cluster/) and Java Treeview (http://jtreeview.sourceforge.net/) as in Shi *et al*., 2012, 2013a, *b*, 2014 b, d). For cluster analysis, all metabolites were quantified as fold change relative to the wild-type bermudagrass plants under control conditions, which was set as 1.0.

### RNA extraction, library construction, and sequencing

For RNA extraction, 28-day-old bermuagrass plants in pots that were irrigated with water or 20 μM melatonin for 7 d were used. Each treatment was represented by two replicate leaf samples, and each sample contained leaves from at least 30 seedlings. Total RNA was extracted with TRIzol (Invitrogen) and was quantified as previously described (Shi *et al.*, 2013*c*). RNA quality was determined using a 2100 Bioanalyzer (Agilent Technologies, Santa Clara, CA, USA) according to the manufacturer’s protocol. The cDNA libraries were constructed with the mRNA-Seq Sample Preparation Kit™ (Illumina, San Diego, CA, USA) and the DNA yield and fragment insert size distribution of each library were determined on the Agilent Bioanalyzer. The cDNA libraries were then sequenced on an Illumina HiSeq2500 sequencing instrument using the 100bp single end protocol.

### Quantitative real-time PCR

The above RNA samples were also used for synthesis of first-strand cDNA with reverse transcriptase (BIO-RAD, Hercules, CA, USA), and the cDNAs were used for quantitative real-time PCR using a CFX96™ Real Time System (BIO-RAD) as previously described (Shi *et al.*, 2013*c*). The specific primers of the analysed genes for real-time PCR are listed in Supplementary Table S1 available at *JXB* online, and the housekeeping genes have been described in Hu *et al.* (2012).

### Bioinformatics analyses of RNA-Seq data

Raw RNA-Seq reads were first trimmed for low quality regions using clean reads with length longer than 25bp, obtained after trimming low quality bases (Q<17) using the SolexQA tool (v2.2) and removing adaptor sequences using the cutadapt tool (v1.3) (Martin, 2011). A total of 679 million clean RNA-Seq reads from 20 libraries, and four libraries were used for transcriptome profiling in this study. Transcriptome analyses of RNA-Seq data were used for transcriptome assembly using Trinity software (v r20131110) (Haas *et al.*, 2013). The resulting pre-assembled transcriptome were refined according to the methods described by Ranjan *et al.* (2014). After transcripts expressed at a low level and redundant sequences were removed, 28 456 high quality transcripts were retained as the final reference transcriptome for bermudagrass.

To obtain putative annotations, the final transcriptome sequences were compared with the NCBI nr protein database by BlastX using an E-value of 1e-5 as the cut-off. Blast2GO (v 2.5.0) (Gotz *et al*., 2008) was used to assign GO terms to each transcript. The final transcriptome sequences were also compared with *Arabidopsis* (TAIR10) and rice (MSU release7) protein database using BlastX with an E-value cut-off of 1e-5. The best hit from these two well-annotated species was used to annotate each bermudagrass transcript.

To evaluate the abundance of each transcript, reads from individual libraries were mapped to the final reference transcripts using Bowtie, and the read counts on each transcript were estimated by the software RSEM with default parameters (Li and Dewey, 2011). Differentially expressed transcripts were identified by the R package edge R (Robinson *et al.*, 2010.).

GO term enrichment analysis of differentially expressed genes was carried out using the topGO Bioconductor package (v 2.16.0) (Alexa *et al.*, 2006) for up- and down-regulated genes, respectively. The Classification SuperViewer Tool (http://bar.utoronto.ca/ntools/cgi-bin/ntools_classification_superviewer.cgi) was used to generate an overview of the enriched pathways (Provart and Zhu, 2003), and MapMan was used as the classification source to assign functional pathways for each gene (Thimm *et al.*, 2004; Yang *et al.*, 2013). The normalized frequency (NF) of each functional category was calculated as described in Chan *et al.* (2011): NF=sample frequency of each category in this experiment/background frequency of each category in the *Arabidopsis* genome.

### Statistical analysis

The experiments in this study were repeated three times and the data shown are the means ±SEs. The means are the average of three independent experiments. Each independent experiment was a pooled sample from at least 30 bermudagrass plants. Bars with different letters above the columns in the figures indicate significant differences at *P*<0.05 (Duncan’s range test).

## Results

### Abiotic stress induced the endogenous melatonin level in bermudagrass

**Fig. 1.**
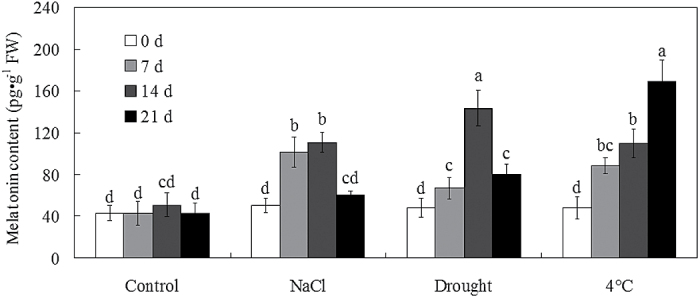
The endogenous melatonin level was induced by salt, drought, and cold stresses in bermudagrass. Twenty-eight-day-old bermudagrass plants were treated with control, 300mM NaCl, drought, and cold (4 °C) stresses for 0, 7, 14, and 21 d, respectively. Bars with different letters above the columns of figures indicate significant differences at *P*<0.05 (Duncan’s range test).

To investigate how abiotic stress affected the melatonin content, endogenous melatonin levels in bermudagrass leaves were quantified after treatments with 300mM NaCl, drought, or cold (4 °C) stresses for 0, 7, 14, and 21 d. Melatonin content remained steady at ~50 pg g^–1^ fresh weight (FW) in non-treated control plants (Fig. 1). After abiotic stress treatments, the melatonin content in bermudagrass leaves significantly increased (Fig. 1). The induction of melatonin content by multiple abiotic stress treatments indicated the *in vivo* role of melatonin in bermudagrass response to abiotic stress.

### Exogenous melatonin improved abiotic stress resistance in bermudagrass

**Fig. 2.**
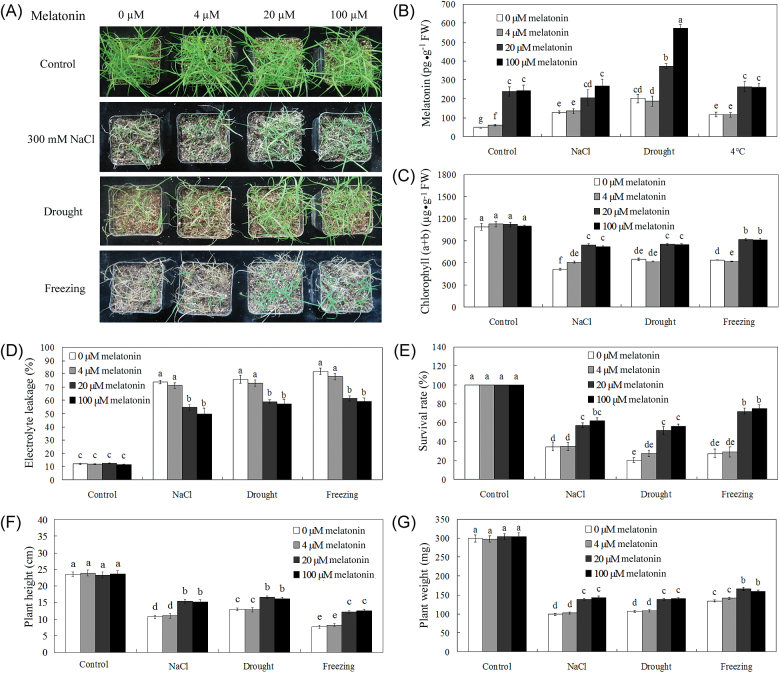
Application of exogenous melatonin improved abiotic stress resistance in bermudagrass. (A) Growth of 28-day-old plants with different melatonin treatments and under control, salt, drought, or freezing stress conditions. The picture is representative of one pot for every treatment in one concentration of melatonin, and at least nine pots with ~120 lines were used for the stress treatments with similar results. (B) Melatonin content of 28-day-old plants with different melatonin treatments and under control, 300mM NaCl, drought stress, and cold (4 °C) stress conditions for 14 d. (C, D) Chlorophyll (C) and EL (D) of 28-day-old plants with different treatments (0, 4, 20, and 100 μM melatonin, respectively) under control, 300mM NaCl, drought stress, and freezing stress conditions for 21 d. (E) The survival rate of bermudagrass plants after 25 d of treatments of control, 300mM NaCl, re-watered from drought, and freezing stresses. (F, G) Plant height (F) and fresh weight (G) of bermudagrass plants after 24 d of treatments of control, 300mM NaCl, re-watered from drought, and freezing stresses. The results shown are the means ±SE (*n*=3 for B, *n*=4 for C–E, and *n*=12 for F and G), and bars with different letters above the columns of figures indicate significant differences at *P*<0.05 (Duncan’s range test). (This figure is available in colour at *JXB* online.)

After 7 d pre-treatment with different concentrations of melatonin (0, 4, 20, and 100 μM melatonin, respectively), no significant differences were observed between non-treated and melatonin pre-treated plants (28 d old) (Fig. 2A). When salt, drought, or cold (4 °C) stresses were applied, the endogenous melatonin levels were activated, and 20 μM and 100 μM melatonin-pre-treated bermudagrass had significantly higher levels than non-melatonin-treated plants (Fig. 2B). Growth and physiological parameters including chlorophyll, EL, survival rate, plant height, and plant biomass (weight) of melatonin-pre-treated 28-day-old bermudagrass plants were generally equivalent to those of non-treated plants under well-watered conditions for the following 25 d (Fig. 2C–G). After salt, drought, or freezing stress treatments, growth of both melatonin-pre-treated and non-treated plants was inhibited, but 20 μM and 100 μM melatonin-pre-treated plants had greener leaf tissues than those of non-treated bermudagrass plants (Fig. 2A). Consistently, 20 μM or 100 μM melatonin-pre-treated plants exhibited a significantly higher chlorophyll content, lower EL, and higher survival rate than did non-treated bermudagrass plants (Fig. 2C–E). Moreover, 20 μM and 100 μM melatonin-pre-treated plants exhibited healthy growth in comparison with non-treated and 4 μM melatonin-pre-treated plants, with significantly higher plant height and weight (Fig. 2F, G). These results indicate that exogenous melatonin application improved salt, drought, and freezing stress resistance in bermudagrass.

### Exogenous melatonin alleviated abiotic stress-induced ROS accumulation in bermudagrass

**Fig. 3.**
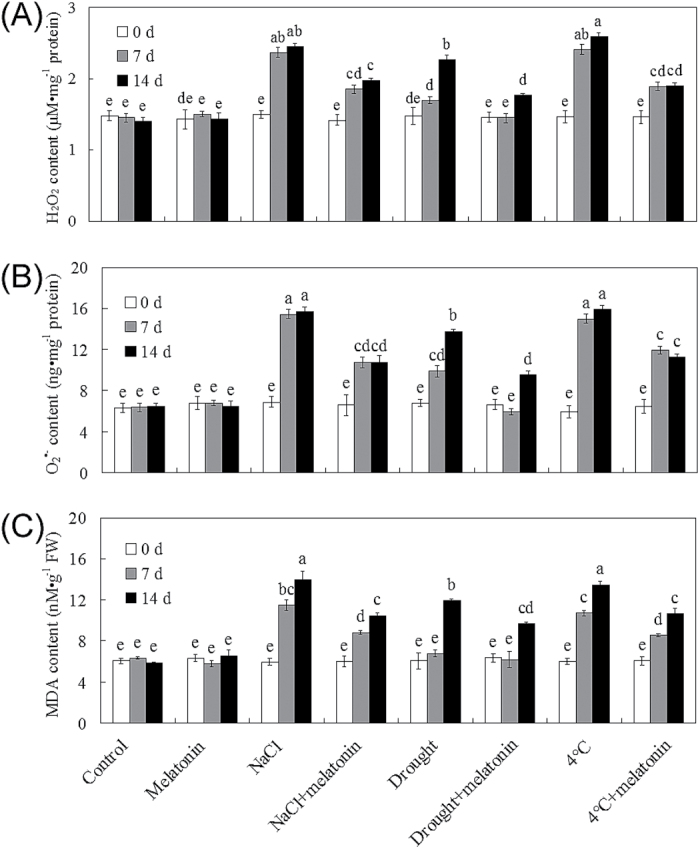
Abiotic stress-induced ROS accumulation and MDA content were alleviated by exogenous melatonin in bermudagrass. (A–C) Quantifications of H_2_O_2_ content (A), O_2_·^–^ content (B), and MDA content (C) of 28-day-old plants with different treatments (control and 20 μM melatonin) under control, 300mM NaCl, drought, and cold (4 °C) stress conditions on the designated days. The results shown are the means ±SEs, and bars with different letters above the columns of figures indicate significant differences at *P*<0.05 (Duncan’s range test).

As the major indicators of the stress-triggered ROS level and oxidative damage, H_2_O_2_, O_2_·^–^, and MDA contents were assayed among control and 20 μM melatonin-pre-treated plants during abiotic stress treatments. Under control conditions, melatonin had no significant effect on H_2_O_2_, O_2_·^–^, and MDA contents (Fig. 3A–C). When salt, drought, and cold (4 °C) stresses were applied, melatonin-pre-treated plants showed significantly lower levels of H_2_O_2_, O_2_·^–^, and MDA in comparison with non-treated bermudagrass plants, conferring less oxidative damage (Fig. 3A–C). These results indicated that exogenous application of melatonin could modulate abiotic stress-triggered ROS accumulation and related oxidative damage in bermudagrass.

### Effects of exogenous melatonin on ROS-related antioxidants in bermudagrass response to abiotic stress

**Fig. 4.**
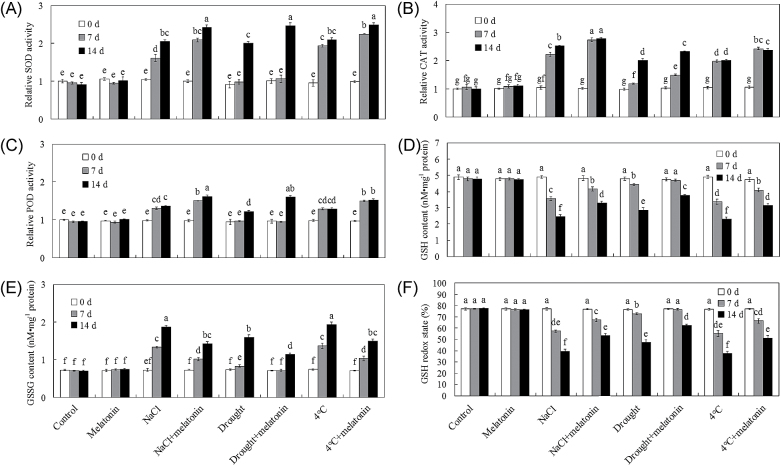
Effects of exogenous melatonin on ROS-related antioxidants in bermudagrass response to abiotic stress. (A–F) Quantifications of SOD activity (A), CAT activity (B), POD activity (C), GSH (D), GSSG (E), and GSH redox state (F) of 28-day-old plants with different treatments (control and 20 μM melatonin) under control, 300mM NaCl, drought, and cold (4 °C) stress conditions on the designated days. The results shown are the means ±SEs, and bars with different letters above the columns of figures indicate significant differences at *P*<0.05 (Duncan’s range test).

To alleviate abiotic stress-triggered ROS burst, plants have developed complex antioxidant defence system, including several antioxidant enzymes and non-enzymatic glutathione antioxidant pool. Under control conditions, no significant differences in antioxidant enzymes and the non-enzymatic glutathione antioxidant pool were found between non-treated and melatonin-pre-treated bermudagrass (Fig. 4A–F). Under abiotic stress conditions, the activities of antioxidant enzymes (SOD, CAT, and POD) and the GSSG content were greatly induced, while GSH content was significantly decreased (Fig. 4A–F). Additionally, melatonin-pre-treated plants showed significantly higher activities of antioxidant enzymes (SOD, CAT, and POD) and a higher GSH redox state in comparison with non-treated plants, conferring more effective antioxidants (Fig. 4A–F). These results indicated that melatonin had significant effects on both antioxidant enzymes and the non-enzymatic glutathione antioxidant pool, which might be consistent with alleviated abiotic stress-induced ROS accumulation and related oxidative damage in bermudagrass.

### Modulation of metabolic homeostasis by exogenous melatonin treatment under control and abiotic stress conditions

**Fig. 5.**
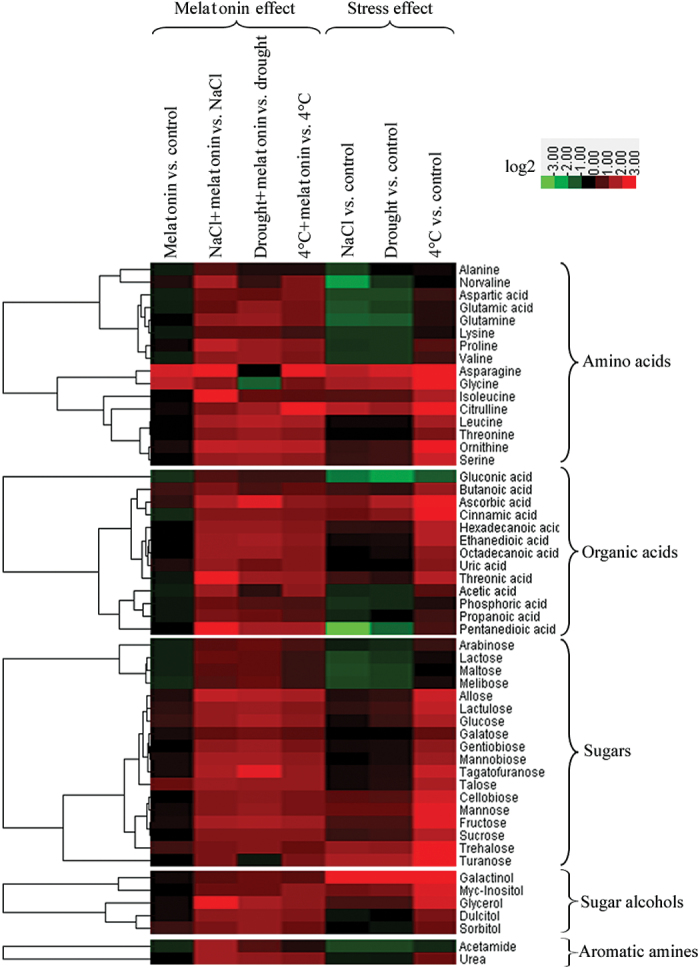
Hierarchical cluster analysis of metabolites modulated by exogenous melatonin in bermudagrass response to abiotic stress. Hierarchical cluster analysis of 54 metabolites of 28-day-old plants by melatonin effect (melatonin versus control, NaCl+melatonin versus NaCl, drought+melatonin versus drought, 4 °C+melatonin versus 4c°C) and by stress effect (NaCl versus control, drought versus control, 4 °C versus control). The log_2_ ratios and scale bars are shown in the resulting tree figure, which was obtained using the CLUSTER software package and the Java Treeview. The concentrations of these metabolites are listed in Supplementary Table S2 at *JXB* online. (This figure is available in colour at *JXB* online.)

**Fig. 6.**
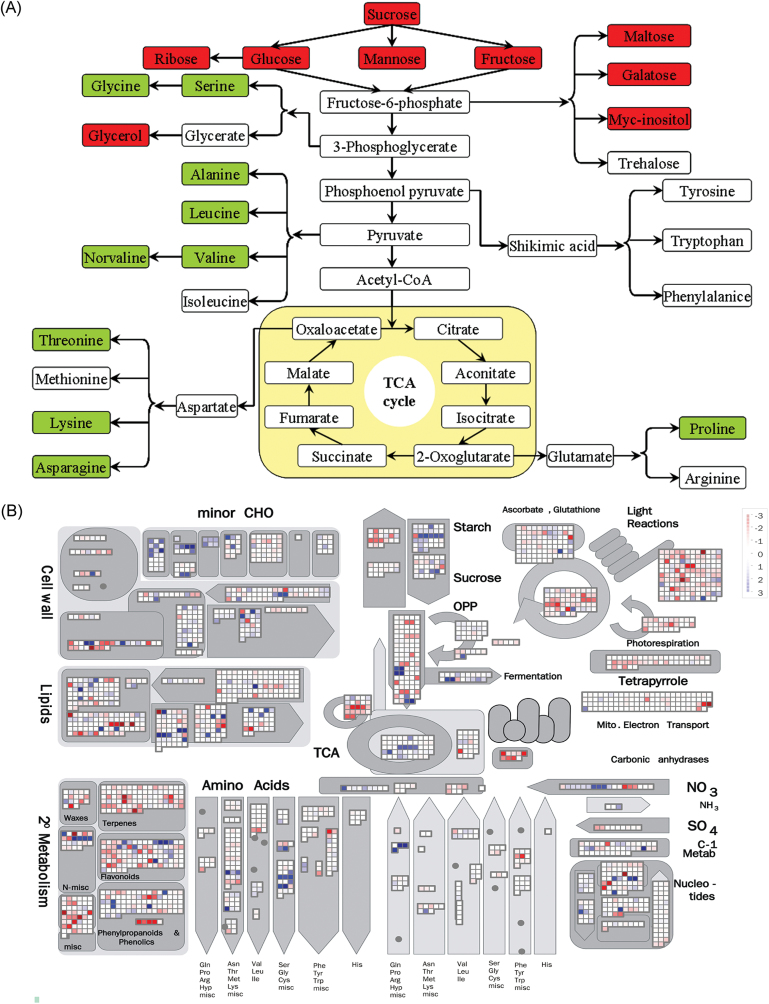
The effects of melatonin on the metabolites that were involved in the carbon metabolic pathway. (A) Assignment of the 18 metabolites from 54 assayed metabolites to the carbon metabolic pathway. The concentrations of these 18 metabolites are shown in Supplementary Table S2 at *JXB* online. (B) Effect of exogenous melatonin treatment on primary metabolism in bermudagrass. Transcripts participating in the same pathway or homologues are represented by a set of closely connected cubes, and the 2-based logarithm of fold change value (log_2_FC) is denoted. (This figure is available in colour at *JXB* online.)

To gain more insights into the modulation of metabolic homeostasis by exogenous melatonin treatment under control and abiotic stress conditions, GC-TOF-MS was performed to identify differentially expressed metabolites. In total, 54 metabolites, comprising 16 amino acids, 13 organic acids, 18 sugars, five sugar alcohols, and two aromatic amines, were reproducibly examined in non-treated and melatonin-pre-treated plants under control and abiotic stress conditions (Fig. 5; Supplementary Table S2 at *JXB* online). Under control conditions, no significant regular pattern of these metabolites was shown in non-treated and melatonin-pre-treated plants (Fig. 5; Supplementary Table S2). When salt, drought, and cold (4 °C) stresses were applied, melatonin-pre-treated plants exhibited higher concentrations of almost all the 54 metabolites than non-treated plants (Fig. 5; Supplementary Table S2). Additionally, many of these metabolites were commonly regulated by salt, drought, and cold (4°C) stresses (Fig. 5; Supplementary Table S2). Interestingly, 18 metabolites, comprising 10 amino acids, six sugars, and two sugar alcohols, were assigned to the carbon metabolic pathway comprising glycolysis, oxidative pentose phosphate pathway, and the tricarboxylic acid (TCA) cycle, indicating the direct link between melatonin and the carbon metabolic pathway in bermudagrass response to abiotic stress. Melatonin-pre-treated plants exhibited significantly higher levels of these metabolites than non-treated plants under abiotic stress conditions (Fig. 6A).

### Transcriptome analysis: GO annotation and enrichment analysis

Since melatonin pre-treatment increased salt, drought, and freezing stress resistance in bermudagrass, the 28-day-old plants (without and with 7 d of pre-treatment) were used for transcriptomic analysis to reveal the effect of melatonin pre-treatment on global transcriptional reprogramming. Approximately 679 million RNA-Seq reads were used for *de novo* assembly of the bermudagrass transcriptome. After removing poorly expressed and redundant transcripts, 28 456 high quality transcripts were retained as the final reference transcriptome. These annotated transcripts were used to search various protein databases. In total, 22 137 (78%) had BLASTX hits in the well-annotated *Arabidopsis* and/or rice protein database (E-value >1e-5). GO annotation was performed using the Blast2GO pipeline, and 18 701 (66%) transcripts were assigned with at least one GO term. Among the three GO categories, 13 402 transcripts were annotated in Biological Process, 14 052 transcripts in Molecular Function, and 14 685 in Cellular Component.

**Fig. 7.**
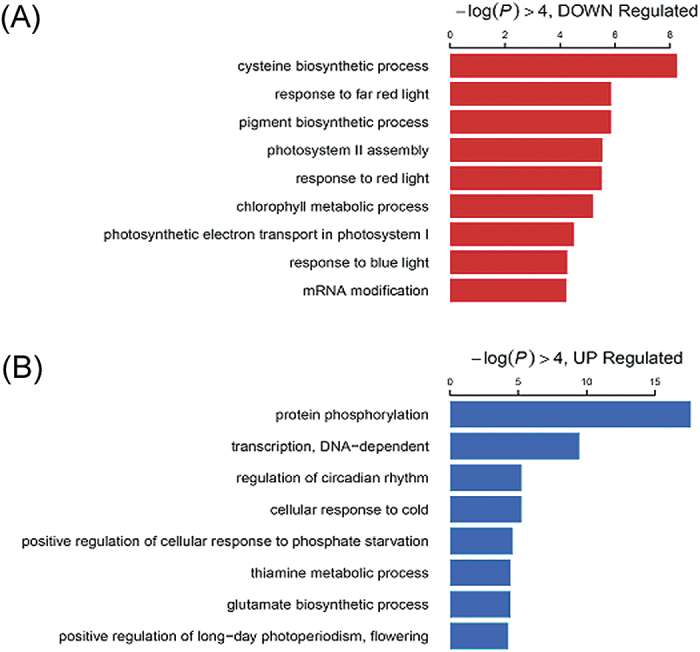
The Biological Process GO terms enrichment of down-regulated (A) and up-regulated (B) genes between control and melatonin-pre-treated bermudagrass. The horizontal axis shows –log10 of the *P*-value. Twenty-one-day-old bermuagrass plants in pots were irrigated with water or 20 μM melatonin for 7 d, then the 28-day-old plants (with and without 7 d of pre-treatment) were used for transcriptomic analysis. (This figure is available in colour at *JXB* online.)

Using fold change >2 and false discovery rate (FDR) <0.05 as thresholds, 3933 transcripts (2361 up-regulated and 1572 down-regulated by exogenous melatonin treatment) were identified as differentially expressed genes (Supplementary Tables S4, S5 at *JXB* online). Many stress-responsive genes were highly induced by exogenous melatonin treatment in bermudagrass (Table 1). Interestingly, several *C-REPEAT-BINDING FACTORS/DEHYDRATION-responsive ELEMENT-BINDING PROTEIN* (*CBF/DREB*) genes and target genes, heat shock transcription factors (TFs), zinc finger TFs, *WRKY*, *MYB*, *bHLH* genes, and hormone-related genes were highly induced >16-fold after melatonin treatment (Table 2). GO enrichment analysis in the biological process domain suggested that genes related to the cysteine biosynthetic process, response to light signal, and the photosynthetic process were down-regulated. In particular, the studies of Wang *et al.* (2012) showed that melatonin can lower ROS damage of many photosynthetic components. Therefore, the expression of genes involved in the photosystem might been suppressed through a negative feedback mechanism. The up-regulated genes were greatly enriched with the GO terms involved in gene expression regulatory process, such as protein phosphorylation, DNA-dependent transcription, regulation of circadian rhythm, etc. (Fig. 7).

**Table 1.** Pathway enrichment analysis of genes whose expression was significantly affected by exogenous melatonin pre-treatment in bermudagrass

Group	MapMan pathway	Up-regulation		Down-regulation	
		NF	*P*-value	NF	*P*-value
I	N metabolism	5.71	**0.0000**	4.00	**0.0140**
	Major CHO metabolism	3.30	**0.0000**	2.31	**0.0100**
	TCA/org transformation	2.58	**0.0023**	2.63	**0.0076**
	Transport	2.05	**0.0000**	2.42	**0.0000**
	Hormone metabolism	2.02	**0.0000**	1.44	**0.0110**
	Metal handling	1.78	**0.0440**	2.19	**0.0260**
	Redox	1.59	**0.0160**	2.60	**0.0000**
	Secondary metabolism	1.42	**0.0094**	2.75	**0.0000**
II	Gluconeogenesis/ Glyoxylate cycle	–	**–**	24.02	**0.0000**
	Glycolysis	–	**–**	2.30	**0.0210**
	Photosystem	–	**–**	8.21	**0.0000**
	Tetrapyrrole synthesis	–	**–**	4.88	**0.0001**
	Fermentation	6.63	**0.0005**	–	**–**
	Minor CHO metabolism	2.99	**0.0000**	–	**–**
	Signalling	2.53	**0.0000**	0.95	0.0550
	C1 metabolism	2.32	**0.0430**	–	**–**
III	RNA	1.84	**0.0000**	1.04	**0.0340**
	Cofactor and vitamin metabolism	1.83	**0.0400**	–	**–**
	Amino acid metabolism	1.71	**0.0036**	1.70	**0.0120**
	Lipid metabolism	1.64	**0.0009**	1.69	**0.0023**
	Nucleotide metabolism	1.54	**0.0280**	1.87	**0.0120**
	Stress	1.46	**0.0000**	–	**–**
	Development	1.41	**0.0016**	–	**–**
	Miscellaneous	1.27	**0.0015**	1.71	**0.0000**
	Protein	1.11	**0.0031**	0.73	0.0000
IV	Cell	0.80	0.0260	–	**–**
	Not assigned	0.45	**0.0000**	0.76	0.0000
	Mitochondrial electron transport/ATP synthesis	0.24	**0.0085**	–	**–**
	DNA	0.12	**0.0000**	0.18	**0.0000**
	MicroRNA, natural antisense, etc	0.00	**0.0000**	0.00	**0.0000**

Twenty-one-day-old bermuagrass plants in pots were irrigated with water or 20 μM melatonin for 7 d, then the 28-day-old plants (without and with 7 d of pre-treatment) were used for transcriptomic analysis. Differentially expressed genes (i.e. with *P*-value ≤0.05 and log_2_ fold change ≥1 or log_2_ fold change ≤ –1) were annotated using the Classification SuperViewer Tool and with MapMan. MapMan was used as the classification source.

Group I indicates highly enriched pathways of both up- and down-regulated genes; group II indicates highly enriched pathways of either up- or down-regulated genes; group III indicates slightly enriched pathways; and group IV indictes under-represented pathways. Scales of normalized frequency (NF) are as follows:

**Table:** 

≥4	2–4	1–20.	5–1	≤0.5

**Table 2.** List of getnes highly induced (>16-fold) by exogenous melatonin pre-treatment in bermudagrass

Seq_ID	logFC	*P*-value	FDR	Putative annoation in *Arabdopsis*	E-value^*a*^	Putative annoation in rice	E-value^*a*^
comp58508_c2_seq12	8.47	5.35E-247	2.50E-245	CBF4, DREB1D	2e-07	DRE	1e-35
comp58508_c2_seq7	7.49	0	0	DREB1A, CBF3	9e-15	DRE	2e-25
comp57631_c0_seq7	2.98	0	0	DREB2A	2e-23	AP2 domain containing protein	2e-62
comp55697_c1_seq4	4.92	0	0	DNA-binding protein	1e-23	AP2 domain containing protein	6e-34
comp49674_c0_seq1	4.49	0	0	HRE1	1e-14	AP2	1e-44
comp52672_c0_seq8	5.57	0	0	ERD15	5e-17	Expressed protein	2e-46
comp51935_c1_seq1	5.89	0	0	Cold-regulated gene 27	3e-13	Expressed protein	4e-74
comp59530_c3_seq1	5.50	4.31E-51	4.22E-50	HSFA6B	5e-61	HSF	4e-133
comp51719_c2_seq14	4.61	0	0	HSFC1	1e-57	HSF	1e-113
comp56606_c0_seq9	5.48	9.30E-52	9.25E-51	HSP20-like	2e-17	hsp20	3e-27
comp60859_c0_seq13	5.13	1.51E-14	5.65E-14	SLT1 | HSP20-like	4e-172	SLT1 protein	0
comp53718_c0_seq3	6.51	0	0	DnaJ	2e-36	Heat shock protein DnaJ	1e-78
comp51393_c1_seq14	5.36	2.40E-58	2.67E-57	DnaJ	6e-16	Heat shock protein DnaJ	1e-36
comp23443_c0_seq1	7.89	6.84E-42	5.67E-41	DNAJ heat shock protein	1e-35	Expressed protein	4e-61
comp53904_c0_seq1	4.32	0	0	WRKY25	4e-35	WRKY53	4e-51
comp55751_c0_seq1	6.18	0	0	WRKY40	2e-53	WRKY71	1e-132
comp55960_c0_seq3	4.57	1.84E-212	7.05E-211	WRKY46	4e-35	WRKY74	1e-93
comp54790_c0_seq12	5.79	1.72E-246	7.99E-245	WRKY51	1e-31	WRKY67	6e-37
comp57307_c2_seq3	5.57	0	0	AZF1	8e-12	C2H2 zinc finger protein	1e-23
comp54697_c5_seq1	4.22	1.63E-163	4.59E-162	ATL6	3e-42	Zinc finger, C3HC4 type	1e-55
comp57307_c1_seq2	5.61	0	0	STZ, ZAT10	4e-25	C2H2 zinc finger protein	3e-36
comp55694_c0_seq3	5.27	0	0	NIP2	1e-51	RING-H2 finger protein ATL2B	2e-90
comp57199_c1_seq6	4.07	1.01E-155	2.69E-154	AtMYB78	6e-14	MYB	2e-14
comp60805_c1_seq6	6.20	0	0	RVE1	5e-36	MYB	7e-97
comp60805_c1_seq4	5.70	0	0	LHY	2e-13	MYB	2e-92
comp58066_c0_seq2	4.59	8.75E-312	5.22E-310	BHLH	3e-38	Ethylene-responsive protein	1e-95
comp54812_c1_seq1	6.33	0	0	BHLH92	3e-19	BHLH	2e-79
comp56171_c3_seq1	5.02	0	0	anac032, NAC032	6e-07	NAC	3e-54
comp54298_c0_seq8	4.56	0	0	AF2	7e-73	NAC	2e-145
comp54697_c3_seq1	4.60	2.23E-132	5.10E-131	RING/U-box protein	1e-28	Zinc finger	4e-49
comp53888_c2_seq1	4.15	9.66E-50	9.21E-49	CMPG2	1e-15	U-box	7e-20
comp53888_c0_seq1	4.74	1.02E-122	2.15E-121	PUB29	2e-31	U-box	9e-90
comp48740_c0_seq1	6.07	0	0	Calcium-binding EF-hand protein	3e-35	EF hand family protein	8e-68
comp48880_c0_seq1	5.33	0	0	Calcium-binding EF-hand protein	1e-28	OsCML31	4e-60
comp61173_c2_seq4	5.64	0	0	Calcium-binding protein	3e-34	OsCML10	1e-57
comp42867_c0_seq1	5.25	6.59E-310	3.93E-308	Calcium-binding protein	1e-43	OsCML14	7e-72
comp50275_c0_seq5	4.36	1.47E-284	7.97E-283	Calcium-binding protein	5e-25	EF hand	1e-40
comp60797_c0_seq1	5.12	0	0	Calmodulin-binding protein	2e-160	Calmodulin-binding protein	0
comp55169_c0_seq2	4.50	0	0	Calmodulin-binding protein	8e-85	Calmodulin-binding protein	3e-145
comp51845_c0_seq1	4.67	0	0	CML43	3e-39	OsCML27	6e-78
comp55740_c0_seq1	4.20	0	0	TCH2, CML24	2e-38	OsCML16	1e-80
comp57565_c0_seq8	9.08	7.50E-220	3.05E-218	CKA1	0	Casein kinase II	0
comp54827_c0_seq1	6.78	6.62E-48	6.09E-47	Kinase	2e-20	Kinase	3e-26
comp55548_c0_seq1	4.39	0	0	Kinase	6e-148	Phosphotransferase	0
comp55703_c1_seq1	4.29	7.18E-159	1.96E-157	Kinase	7e-65	Tyrosine protein kinase	2e-124
comp60546_c0_seq1	4.11	1.75E-75	2.41E-74	Kinase	0	Leucine-rich repeat protein	0
comp54489_c2_seq1	5.52	3.60E-185	1.19E-183	JMJD5	8e-139	jmjC protein 5	9e-146
comp48478_c1_seq1	4.45	1.09E-76	1.52E-75	HDA18	2e-64	Histone deacetylase	2e-71
comp57103_c1_seq1	4.45	0	0	ERF-1	3e-22	ERF	1e-43
comp51949_c0_seq2	4.49	0	0	JAZ2, TIFY10B	2e-10	ZIM protein	8e-31
comp59913_c1_seq8	7.52	0	0	CYP707A1	8e-50	Cytochrome P450	1e-64
comp50455_c0_seq1	3.42	0	0	PYL5, RCAR8	7e-55	Cyclase/dehydrase	1e-87
comp55459_c2_seq1	4.20	0	0	PP2C	6e-101	PP2C	1e-164
comp55059_c0_seq4	4.33	2.77E-269	1.43E-267	SHY2, IAA3	2e-47	OsIAA24	2e-64
comp56859_c0_seq6	5.51	3.14E-169	9.21E-168	Alcohol dehydrogenase	8e-100	Dehydrogenase	9e-100
comp55475_c1_seq1	4.41	0	0	WCRKC thioredoxin 1	4e-45	Thioredoxin	4e-80
comp49522_c0_seq2	4.02	9.96E-36	7.38E-35	Oxidoreductase	2e-88	Dehydrogenase	4e-136
comp57222_c0_seq2	5.09	0	0	FMO1	2e-91	Monooxygenase	0
comp56296_c0_seq6	5.02	2.03E-49	1.92E-48	Copper transport protein	2e-09	Heavy metal-associated protein	3e-26
comp46851_c0_seq1	4.92	5.38E-117	1.07E-115	Heavy metal transport	1e-06	Expressed protein	4e-21
comp51430_c0_seq1	4.45	7.94E-278	4.22E-276	Heavy metal transport	1e-15	Heavy metal-associated protein	3e-41

Twenty-one-day-old bermuagrass plants in pots were irrigated with water or 20 μM melatonin for 7 d, then the 28-day-old plants (with and without 7 d of pre-treatment) were used for transcriptomic analysis.

logFC, log2 fold change; FDR, false discovery rate.

^*e*^E-value, expected value for putative annotation in *Arabidopsis* or rice.

To confirm the reliability of the RNA-Seq data, the expression of 18 genes (nine up-regulated and nine down-regulated by exogenous melatonin treatment) that were differentially expressed between control and melatonin-treated plants was assessed via quantitative real-time PCR. Consistently, the results of the real-time PCR assay exhibited the same trend and were correlated with the RNA-Seq data (Supplementary Fig. S1 at *JXB* online), confirming the reproducibility of RNA-Seq data.

### Pathway and GO term enrichment analyses

The transcriptome data were submitted to the Mercator web tool to align with the public protein database, and 14 288 transcripts were located in at least one point in plant biological pathways. As shown in Table 1, pathway analysis revealed that melatonin affected the expression of many genes involved in N metabolism, minor carbohydrate metabolism, TCA/org transformation, transport, hormone metabolism, metal handling, redox, and secondary metabolism (Table 1, group I). Other transcripts involved in stress response and metabolism were extensively changed after melatonin treatment (Fig. 6B; Supplementary Fig. S2 at *JXB* online). These results indicated that melatonin treatment might induce a stress response in bermudagrass. The pathway analysis results were consistent with the study carried out by Weeda *et al.* (2014), which showed that melatonin altered many genes involved in plant defence (Supplementary Fig. S2), and these changes might contribute to the enrichment of stress-related GO terms (Fig. 7).

## Discussion

As sessile organisms, plants have developed sophisticated strategies to respond to diverse environmental stresses. The stress signals are perceived by several receptors at the cell membrane level, followed by their transduction to multiple second messengers such as abscisic acid (ABA), H_2_O_2_, nitric oxide (NO), etc. These activate downstream stress-responsive genes and physiological responses, eventually leading to protective responses at the whole-plant level (Shi *et al*., 2012, 2013a, *b*, 2014 b, c, d; Shi and Chan, 2014*a*, b). Although no direct evidence had indicated that melatonin could serve as a second messenger, the induction of melatonin by multiple stress treatments (Fig. 1) indicates an *in vivo* role for melatonin in bermudagrass response to abiotic stress (Arnao and Hernández-Ruiz, 2006, 2009*b*, 2013*a*, b; Tan *et al.*, 2007*a*, 2012).

In this study, the protective role of melatonin on the response of bermudagrass to abiotic stress was revealed. Under control conditions, melatonin had no significant effect on bermudagrass growth or its physiological responses (Fig. 2). Under abiotic stress conditions, however, melatonin-pre-treated plants exhibited significantly higher chlorophyll content, lower EL, and higher survival rate than did non-treated bermudagrass plants (Fig. 2C–E). After recovery from abiotic stress treatments, melatonin-pre-treated plants exhibited better growth status than non-treated plants, with higher biomass (plant height and weight) (Fig. 2F, G). These results indicate that exogenous melatonin application improved salt, drought, or freezing stress resistance in bermudagrass, in accordance with the enhanced resistance to cold stress (Posmyk *et al.*, 2009*a*; Kang *et al.*, 2010; Bajwa *et al.*, 2014), copper stress (Posmyk *et al.*, 2008, 2009b), high temperature (Byeon and Back, 2014*b*), salt stress (Li *et al.*, 2012), osmotic stress (Zhang *et al.*, 2013), drought stress (Wang *et al.*, 2014), and pathogen infection (Yin *et al.*, 2013) due to melatonin in various plant species.

As an antioxidant in animals, melatonin scavenges free radicals directly, stimulates the activities of antioxidant enzymes including CAT, SOD (both MnSOD and CuSOD), glutathione reductase (GR), and glutathione peroxidase (GPX), and increases the efficiency of mitochondrial oxidative phosphorylation (Tan *et al*., 1993, 1999, 2003, 2007a, b; Reiter *et al.*, 2000; Kolář and Macháčkova, 2005). In plants, melatonin is also an important antioxidant and a radical scavenger (Arnao *et al.*, 1996, 2001; Cano *et al.*, 2003, 2006). Li *et al.* (2012) also found that exogenous melatonin modulates salinity-induced oxidative damage by directly scavenging H_2_O_2_ and enhancing the activities of antioxidative enzymes in *Malus hupehensis*. Consistently, exogenous application of melatonin significantly activated ROS detoxification of antioxidants, including enzymatic antioxidant enzymes (SOD, CAT, and POD) and non-enzymatic glutathione (GSH redox state), to maintain cellular ROS (mainly including H_2_O_2_ and O_2_·^–^) at a relatively low level. This results in the alleviation of abiotic stress-induced oxidative damage and further conferred improved abiotic stress resistance (Figs 3, 4). Consistently, RNA-Seq found that many genes involved in redox, many *POD* genes and *glutathione S-transferase*s (*GST*) genes were significantly modulated by exogenous melatonin treatment (Table 1; Supplementary Fig. S2 at *JXB* online). In summary, the positive modulation by exogenous melatonin of the ROS detoxification system might contribute greatly to enhanced abiotic stress resistance in bermudagrass.

Moreover, comparative metabolomic analysis showed the actions of melatonin treatment on carbon metabolites and amino acid metabolism under abiotic stress conditions. Notably, melatonin-pre-treated plants exhibited higher concentrations of 54 metabolites compared with non-melatonin-treated plants (Fig. 5; Supplementary Table S2 at *JXB* online). Among these metabolites, proline and some carbohydrates (fructose, sucrose, glucose, maltose, cellobiose, trehalose, galactose, and galactinol) are important compatible solutes to respond to abiotic stress for osmotic adaptation (Krasensky and Jonak, 2012). Thus, higher levels of proline and carbohydrates (glucose, maltose, fructose, sucrose, and trehalose) in melatonin-pre-treated plants provided beneficial effects under abiotic stress conditions. In addition, higher levels of other metabolites including multiple amino acids, organic acids, and sugars in melatonin-pre-treated plants indicate the beneficial physiological processes in melatonin-pre-treated plants during abiotic stress treatment; the data further confirm the protective role of melatonin in response to abiotic stress. Notably, 18 metabolites comprising 10 amino acids, six sugars, and two sugar alcohols of the carbon metabolic pathway exhibited significantly higher levels in melatonin-pre-treated plants under abiotic stress conditions (Fig. 6A). These results indicate the widespread effects of melatonin treatment in carbon metabolism and amino acid metabolism; these metabolites might play some role in melatonin-mediated abiotic stress resistance in bermudagrass.

Additionally, comparative transcriptomic analysis identified 2361 up-regulated and 1572 down-regulated transcripts as a consequence of exogenous melatonin treatment. Quantitative real-time PCR of 18 genes supported the reliability of the RNA-Seq data (Supplementary Fig. S1 at *JXB* online). Pathway enrichment analysis indicated that eight pathways were over-represented among differentially expressed genes between control and melatonin-treated bermudagrass plants, including N metabolism, major carbohydrate metabolism, TCA/org transformation, transport, hormone metabolism, metal handling, redox, and secondary metabolism (Table 1, group I). The enrichment of redox-related genes affected by melatonin (Table 1, group I) was consistent with the effects of exogenous melatonin on ROS detoxification in bermudagrass (Figs 4, 5). In animals, melatonin is known to be involved in circadian rhythms (Tal *et al.*, 2011). Interestingly, the GO enrichment analysis also showed that transcripts that function in regulation of rhythms and flowering were over-represented (Fig. 7). Notably, the pathway analysis results were consistent with those of the study carried out in *Arabidopsis* by Weeda *et al.* (2014). Thus, melatonin altered many genes involved in plant defence in bermudagrass (Supplementary Fig. S2); these changes probably contributed to the enrichment of stress-related GO terms (Fig. 7). Additionally, exogenous melatonin treatment had significant effects on various signalling pathways including primary metabolism, secondary metabolism, photosynthesis, large enzyme families, receptor-like kinases, proteolysis, and autophagy pathways in bermudagrass as determined using MapMan software (Fig. 6B; Supplementary Fig. S2). Those genes modulated by exogenous melatonin treatment might also contribute to melatonin-enhanced abiotic stress resistance in bermudagrass.

Asparagine accumulation shows that nitrogen re-distribution and mobilization were important features of the melatonin response (Lea *et al.*, 2007; Maaroufi-Dguimi *et al.*, 2011). Jia *et al.* (2001) also suggested that asparagine may be a signalling molecule involved in sensing the nitrogen status. In addition, asparagine is an amino group donor for the synthesis of the photorespiratory intermediate glycine. Nagy *et al.* (2013) and Shi *et al.* (2014) found that this is also a good indicator of drought stress in drought-tolerant and sensitive wheat and bermudagrass cultivars. At the same time, some carbohydrate metabolism compounds increased: these included fructose, glucose, and trehalose, but not sucrose. Such differential dynamics of carbohydrates could reflect modifications of carbon balance and carbon utilization. Moreover, asparagine synthetase genes that are involved in asparagine synthesis are regulated by the level of carbohydrates (Lam *et al.*, 1998; Foito *et al.*, 2009). TCA/org transformation is important for the Calvin cycle for CO_2_ assimilation and separation of initial carbon fixation by contact with air and secondary carbon fixation into sugars (Selwood and Jaffe, 2011). Glycolysis is an important metabolic pathway in carbohydrate metabolism, and the central role of glycolysis in the plant metabolic pathway is to provide energy such as ATP and generates precursors for anabolism such as fatty acids and amino acids (Plaxton, 1996). In accordance with the metabolic profiles, transcriptomic analysis found that many genes involved in TCA/org transformation and N metabolism were modulated by melatonin treatment (Table 1; Fig. 6B). Genes which functioned in sucrose and amino acid metabolism were also greatly changed after melatonin treatment (Fig. 6B), leading to altered sucrose and amino acid contents revealed by metabolite analysis (Figs 5, 6). These results showed that the underlying mechanisms related to melatonin may involve major reorientation of photorespiratory and carbohydrate and nitrogen metabolism.

To date, various TFs have been shown to be involved in plant stress responses via activating stress-responsive gene expression, such as BASIC LEUCINE ZIPPER PROTEINS (bZIPs), CBF/DREBs, ETHYLENE-RESPONSIVE ELEMENT-BINDING FACTORS (ERFs), MYBs, WRKYs, and zinc finger proteins (ZFPs) (Shi *et al*., 2014*a*, *e*). In the current study, many TFs were significantly regulated by exogenous melatonin treatment (Table 2; Supplementary Tables S4, S5 at *JXB* online), and these TFs might contribute to the enhanced stress tolerance of melatonin-treated plants, thus indicating that melatonin might pre-condition to be resistant to abiotic stresses. Some protein kinases [such as mitogen-activated protein kinase (MAPK)] and calcium signalling kinases [including calcium-dependent protein kinases (CDPKs), calcineurin B-like (CBL)-interacting protein kinases (CIPKs), and calcium-related protein kinases (CRKs)] were also transcriptionally regulated by exogenous melatonin treatment (Table 1; Supplementary Tables S4, S5). This suggests that kinase signalling might play critical roles in melatonin-mediated stress responses. As sessile organisms, plants cannot avoid unfavourable stress conditions by adjusting their location; thus, they have evolved complex strategies to perceive stress signals and further translate the perception into effective responses, which might largely depend on various protein kinases and TFs (Shi *et al*., 2014*a*, *e*).

Recently, it was found that one cysteine2/histidine2-type zinc finger TF, zinc finger of *Arabidopsis thaliana 6* (*ZAT6*), is involved in melatonin-mediated freezing stress response, and the AtZAT6-activated *CBF* pathway was essential for melatonin-mediated freezing stress response in *Arabidopsis* (Shi and Chan, 2014*a*). This study together with others in sunflower (*Helianthus annuus*) (Mukherjee *et al.*, 2014) and in *Arabidopsis* (Shi and Chan, 2014*a*; Weeda *et al.*, 2014) indicate that melatonin is involved in long-distance signal transduction in plants. Moreover, some important genes in plant hormone signalling [*RCAR/PYR/PYL*, *SNF1-related protein kinases 2* (*SnRK2*), and *nine-cis-epoxycarotenoid dioxygenase* (*NCED*) genes in ABA signalling, and jasmonate (JA)-JIM-domain proteins (JAZs) in JA signalling] that were significantly regulated by exogenous melatonin treatment (Table 1; Supplementary Tables S4, S5 at *JXB* online) might also have some function in melatonin-mediated cross-talk among plant hormones, as well as in stress responses. Kolář and Macháčkova (2005) also found that melatonin might function as an auxin to promote vegetative growth. These results suggested that melatonin might serve as a plant hormone that cross-talks with other plant hormones. Thus, melatonin triggered extensive transcriptional reprogramming and pre-conditioned resistance to multiple abiotic stresses. Further investigation of the *in vivo* roles of these genes will shed additional light on melatonin-mediated stress responses in bermudagrass.

Taken together, this study provides the first evidence of the protective roles of exogenous melatonin in bermudagrass response to multiple abiotic stresses. This involved the activation of antioxidants, modulation of metabolic homeostasis, and extensive transcriptional reprogramming.

## Supplementary data

Supplementary data are available at *JXB* online.

Figure S1. Validation of differentially expressed genes by quantitative real-time PCR.

Figure S2. Effect of exogenous melatonin treatment on stress-related pathways in bermudagrass.

Table S1. The specific primers used for real-time PCR.

Table S2. Concentrations of 54 metabolites in 28-day-old bermudagrass plants under control conditions and different treatments [20 μM melatonin, 300mM NaCl, drought, and cold (4 °C)] stress conditions for 14 d.

Table S3. Summary of RNA-Seq data.

Table S4. List of up-regulated genes in melatonin-treated bermudagrass plants.

Table S5. List of down-regulated genes in melatonin-treated bermudagrass plants.

Supplementary Data

## References

[CIT0001] Alexa A Rahnenfuhrer J Lengauer T 2006 Improved scoring of functional groups from gene expression data by decorrelating GO graph structure. Bioinformatics 22, 1600–1607. 16606683 10.1093/bioinformatics/btl140

[CIT0002] Arnao MB Cano A Alcolea JF Acosta M 2001 Estimation of free radical-quenching activity of leaf pigment extracts. Phytochemical Analysis 12, 138–143. 11705243 10.1002/pca.571

[CIT0003] Arnao MB Hernández-Ruiz J 2006 The physiological function of melatonin in plants. Plant Signaling and Behavior 1, 89–95. 19521488 10.4161/psb.1.3.2640 PMC2635004

[CIT0004] Arnao MB Hernández-Ruiz J 2007 Melatonin in plants: more studies are necessary. Plant Signaling and Behavior 2, 381–382. 19704606 10.4161/psb.2.5.4260 PMC2634219

[CIT0005] Arnao MB Hernández-Ruiz J 2009a Protective effect of melatonin against chlorophyll degradation during the senescence of barley leaves. Journal of Pineal Research 46, 58–63. 18691358 10.1111/j.1600-079X.2008.00625.x

[CIT0006] Arnao MB Hernández-Ruiz J 2009b Chemical stress by different agents affects the melatonin content of barley roots. Journal of Pineal Research 46, 295–299. 19196434 10.1111/j.1600-079X.2008.00660.x

[CIT0007] Arnao MB Hernández-Ruiz J 2013a Growth conditions determine different melatonin levels in *Lupinus albus* L. Journal of Pineal Research 55, 149–155. 23600673 10.1111/jpi.12055

[CIT0008] Arnao MB Hernández-Ruiz J 2013b Growth conditions influence the melatonin content of tomato plants. Food Chemistry 138, 1212–1214. 23411233 10.1016/j.foodchem.2012.10.077

[CIT0009] Arnao MB Sanchez-Bravo J Acosta M 1996 Indole-3-carbinol as a scavenger of free radicals. Biochemistry and Molecular Biology International 39, 1125–1134. 8876965 10.1080/15216549600201302

[CIT0010] Bajwa VS Shukla MR Sherif SM Murch SJ Saxena PK 2014 Role of melatonin in alleviating cold stress in *Arabidopsis thaliana* . Journal of Pineal Research 56, 238–245. 24350934 10.1111/jpi.12115

[CIT0011] Byeon Y Back K 2014a An increase in melatonin in transgenic rice causes pleiotropic phenotypes, including enhanced seedling growth, delayed flowering, and low grain yield. Journal of Pineal Research 56, 408–414. 24571270 10.1111/jpi.12129

[CIT0012] Byeon Y Back K 2014b Melatonin synthesis in rice seedlings *in vivo* is enhanced at high temperatures and under dark conditions due to increased serotonin *N*-acetyltransferase and *N*-acetylserotoni*n* methyltransferase activities. Journal of Pineal Research 56, 189–195. 24313332 10.1111/jpi.12111

[CIT0013] Byeon Y Park S Kim YS Back K 2013 Microarray analysis of genes differentially expressed in melatonin-rich transgenic rice expressing a sheep serotonin N-acetyltransferase. Journal of Pineal Research 55, 357–363. 23889160 10.1111/jpi.12077

[CIT0014] Byeon Y Park S Kim YS Park DH Lee S Back K 2012 Light-regulated melatonin biosynthesis in rice during the senescence process in detached leaves. Journal of Pineal Research 53, 107–111. 22289080 10.1111/j.1600-079X.2012.00976.x

[CIT0015] Byeon Y Park S Lee HY Kim YS Back K 2014 Elevated production of melatonin in transgenic rice seeds expressing rice tryptophan decarboxylase. Journal of Pineal Research 56, 275–282. 24433490 10.1111/jpi.12120

[CIT0016] Calvo JR González-Yanes C Maldonado MD 2013 The role of melatonin in the cells of the innate immunity: a review. Journal of Pineal Research 55, 103–120. 23889107 10.1111/jpi.12075

[CIT0017] Cano A Hernández-Ruiz J Arnao MB 2006 Changes in hydrophilic antioxidant activity in *Avena sativa* and *Triticum aestivum* leaves of different age during de-etiolation and high-light treatment. Journal of Pineal Research 119, 321–327. 10.1007/s10265-006-0275-1 16628378

[CIT0018] Cano A Alcaraz O Arnao MB 2003 Free radical-scavenging activity of indolic compounds in aqueous and ethanolic media. Analytical and Bioanalytical Chemistry 376, 33–37. 12734615 10.1007/s00216-003-1848-7

[CIT0019] Chan Z Grumet R Loescher W 2011 Global gene expression analysis of transgenic, mannitol-producing, and salt-tolerant *Arabidopsis thaliana* indicates widespread changes in abiotic and biotic stress-related genes. Journal of Experimental Botany 62, 4787–4803. 21821598 10.1093/jxb/err130 PMC3192998

[CIT0020] Dubbels R Reiter RJ Klenke E Goebel A Schnakenberg E Ehlers C Schiwara HW Schloot W 1995 Melatonin in edible plants identified by radioimmunoassay and by high performance liquid chromatography-mass spectrometry. Journal of Pineal Research 18, 28–31. 7776176 10.1111/j.1600-079x.1995.tb00136.x

[CIT0021] Foito A Byrne SL Shepherd T Stewart D Barth S 2009 Transcriptional and metabolic profiles of *Lolium perenne* L. genotypes in response to a PEG-induced water stress. Plant Biotechnology Journal 7, 719–732. 19702648 10.1111/j.1467-7652.2009.00437.x

[CIT0022] Galano A Tan DX Reiter RJ 2011 Melatonin as a natural ally against oxidative stress: a physicochemical examination. Journal of Pineal Research 51, 1–16. 21752095 10.1111/j.1600-079X.2011.00916.x

[CIT0023] Götz S García-Gómez JM Terol J Williams TD Nagaraj SH Nueda MJ Robles M Talón M Dopazo J Conesa A 2008 High-throughput functional annotation and data mining with the Blast2GO suite. Nucleic Acids Research 36, 3420–3435. 18445632 10.1093/nar/gkn176 PMC2425479

[CIT0024] Haas BJ Papanicolaou A Yassour M 2013 De novo transcript sequence reconstruction from RNA-seq using the Trinity platform for reference generation and analysis. Nature Protocols 8, 1494–1512. 10.1038/nprot.2013.084 PMC3875132 23845962

[CIT0025] Hattori A Migitaka H Iigo M Itoh M Yamamoto K Ohtani-Kaneko R Hara M Suzuki T Reiter RJ 1995 Identification of melatonin in plants and its effects on plasma melatonin levels and binding to melatonin receptors in vertebrates. Biochemistry and Molecular Biology International 35, 627–634. 7773197

[CIT0026] Hernández-Ruiz J Arnao MB 2008 Distribution of melatonin in different zones of lupin and barley plants at different ages in the presence and absence of light. Journal of Agricultural and Food Chemistry 56, 10567–10573. 18975965 10.1021/jf8022063

[CIT0027] Hernández-Ruiz J Cano A Arnao MB 2005 Melatonin acts as a growth-stimulating compound in some monocot species. Journal of Pineal Research 39, 137–142. 16098090 10.1111/j.1600-079X.2005.00226.x

[CIT0028] Hu L Huang Z Liu S Fu J 2012 Growth response and gene expression in antioxidant related enzymes in two bermudagrass genotypes differing in salt tolerance. Journal of the American Society of Horticultural Science 137, 134–143.

[CIT0029] Jia M Keutgen N Matsuhashi S Mitzuniwa C Ito T Fujimura T Hashimoto S 2001 Ion chromatographic analysis of selected free amino acids and cations to investigate the change of nitrogen metabolism by herbicide stress in soybean (*Glycine max*). Journal of Agricultural and Food Chemistry 49, 276–280. 11305252 10.1021/jf990344c

[CIT0030] Kang K Lee K Park S Kim YS Back K 2010 Enhanced production of melatonin by ectopic overexpression of human serotonin N-acetyltransferase plays a role in cold resistance in transgenic rice seedlings. Journal of Pineal Research 49, 176–182. 20586889 10.1111/j.1600-079X.2010.00783.x

[CIT0031] Kolář J Macháčkova I 2005 Melatonin in higher plants: occurrence and possible functions. Journal of Pineal Research 39, 333–341. 16207287 10.1111/j.1600-079X.2005.00276.x

[CIT0032] Krasensky J Jonak C 2012 Drought, salt, and temperature stress-induced metabolic rearrangements and regulatory networks. Journal of Experimental Botany 63, 1593–1608. 22291134 10.1093/jxb/err460 PMC4359903

[CIT0033] Lam HM Hsieh MH Coruzzi G 1998 Reciprocal regulation of distinct asparagine synthetase genes by light and metabolites in *Arabidopsis thaliana* . The Plant Journal 16, 345–353. 9881155 10.1046/j.1365-313x.1998.00302.x

[CIT0034] Lea US Slimestad R Smedvig P Lillo C 2007 Nitrogen deficiency enhances expression of specific MYB and bHLH transcription factors and accumulation of end products in the flavonoid pathway. Planta 225, 1245–1253. 17053893 10.1007/s00425-006-0414-x

[CIT0035] Li B Dewey CN 2011 RSEM: accurate transcript quantification from RNA-Seq data with or without a reference genome. BMC Bioinformatics 12, 323. 21816040 10.1186/1471-2105-12-323 PMC3163565

[CIT0036] Li C Wang P Wei Z Liang D Liu C Yin L Jia D Fu M Ma F 2012 The mitigation effects of exogenous melatonin on salinity-induced stress in *Malus hupehensis* . Journal of Pineal Research 53, 298–306. 22507106 10.1111/j.1600-079X.2012.00999.x

[CIT0037] Lisec J Schauer N Kopka J Willmitzer L Fernie AR 2006 Gas chromatography mass spectrometry-based metabolite profiling in plants. Nature Protocols 1, 387–396. 10.1038/nprot.2006.59 17406261

[CIT0038] Maaroufi-Dguimi H Debouba M Gaufichon L Clément G Gouia H Hajjaji A Suzuki A 2011 An Arabidopsis mutant disrupted in ASN2 encoding asparagine synthetase 2 exhibits low salt stress tolerance. Plant Physiology and Biochemistry 49, 623–628. 21478030 10.1016/j.plaphy.2011.03.010

[CIT0039] Martin M 2011 Cutadapt removes adapter sequences from high-throughput sequencing reads. EMBnet Journal 17, 10–12.

[CIT0040] Manchester LC Tan DX Reiter RJ Park W Monis K Qi W 2000 High levels of melatonin in the seeds of edible plants: possible function in germ tissue protection. Life Sciences 67, 3023–3029. 11125839 10.1016/s0024-3205(00)00896-1

[CIT0041] Mukherjee S David A Yadav S Baluška F Bhatla SC 2014 Salt stress-induced seedling growth inhibition coincides with differential distribution of serotonin and melatonin in sunflower seedling roots and cotyledons. Physiologia Plantarum 152, 714–728. 24799301 10.1111/ppl.12218

[CIT0042] Murch SJ Alan AR Cao J Saxena PK 2009 Melatonin and serotonin in flowers and fruits of *Datura metel* L. Journal of Pineal Research 47, 277–283. 19732299 10.1111/j.1600-079X.2009.00711.x

[CIT0043] Nagy Z Németh E Guóth A Bona L Wodala B Pécsváradi A 2013 Metabolic indicators of drought stress tolerance in wheat: glutamine synthetase isoenzymes and Rubisco. Plant Physiology and Biochemistry 67, 48–54. 23542183 10.1016/j.plaphy.2013.03.001

[CIT0044] Okazaki M Ezura H 2009 Profiling of melatonin in model tomato (*Solanum lycopersicum* L.) cultivar Micro-Tom. Journal of Pineal Research 43, 338–343. 19317796 10.1111/j.1600-079X.2009.00668.x

[CIT0045] Okazaki M Higuchi K Aouni A Ezura H 2010 Lowering intercellular melatonin by transgenic analysis of indoleamine 2,3-dioxygenase from rice in tomato plants. Journal of Pineal Research 49, 239–247. 20609074 10.1111/j.1600-079X.2010.00788.x

[CIT0046] Okazaki M Higuchi K Hanawa Y Shiraiwa Y Ezura H 2009 Cloning and characterization of a *Chlamydomonas reinhardtii* cDNA arylalkylamine *N* -acetyltransferase and its use in the genetic engineering of melatonin content in the Micro-Tom tomato. Journal of Pineal Research 43, 373–382. 19552760 10.1111/j.1600-079X.2009.00673.x

[CIT0047] Pape C Lüning K 2006 Quantification of melatonin in phototrophic organisms. Journal of Pineal Research 41, 157–165. 16879322 10.1111/j.1600-079X.2006.00348.x

[CIT0048] Park S Lee DE Jang H Byeon Y Kim YS Back K 2013 Melatonin-rich transgenic rice plants exhibit resistance to herbicide-induced oxidative stress. Journal of Pineal Research 54, 258–263. 22856683 10.1111/j.1600-079X.2012.01029.x

[CIT0049] Pelagio-Flores R Muňoz-Parra E Ortiz-Castro R López-Bucio J 2012 Melatonin regulates Arabidopsis root system architecture likely acting independently of auxin signaling. Journal of Pineal Research 53, 279–288. 22507071 10.1111/j.1600-079X.2012.00996.x

[CIT0050] Plaxton WC 1996 The organization and regulation of plant glycolysis. Annual Review of Plant Biology and Plant Molecular Biology 47, 185–214. 10.1146/annurev.arplant.47.1.185 15012287

[CIT0051] Posmyk MM Bałabusta M Wieczorek M Sliwinska E Janas KM 2009a Melatonin applied to cucumber (*Cucumis sativus* L.) seeds improves germination during chilling stress. Journal of Pineal Research 46, 214–223. 19141087 10.1111/j.1600-079X.2008.00652.x

[CIT0052] Posmyk MM Janas KM Kontek R 2009b Red cabbage anthocyanin extract alleviates copper-induced cytological disturbances in plant meristematic tissue and human lymphocytes. Biometals 22, 479–490. 19152114 10.1007/s10534-009-9205-8

[CIT0053] Posmyk MM Kuran H Marciniak K Janas KM 2008 Presowing seed treatment with melatonin protects red cabbage seedlings against toxic copper ion concentrations. Journal of Pineal Research 45, 24–31. 18205729 10.1111/j.1600-079X.2007.00552.x

[CIT0054] Provart N Zhu T 2003 A browser-based functional classification superviewer for Arabidopsis genomics. Current Computer Molecular Biology 2003, 271–272.

[CIT0055] Ramakrishna A Giridhar P Sankar KU Ravishankar GA 2012 Melatonin and serotonin profiles in beans of Coffea species. Journal of Pineal Research 52, 470–476. 22017393 10.1111/j.1600-079X.2011.00964.x

[CIT0056] Ranjan A Ichihashi Y Farhi M Zumstein K Townsley B David-Schwartz R Sinha NR 2014 De novo assembly and characterization of the transcriptome of the parasitic weed *Cuscuta pentagona* identifies genes associated with plant parasitism. Plant Physiology 166, 1186–1199. 24399359 10.1104/pp.113.234864 PMC4226353

[CIT0057] Reiter RJ Tan DX Burkhardt S Manchester LC 2001 Melatonin in plants. Nutrition Reviews 59, 286–290. 11570431 10.1111/j.1753-4887.2001.tb07018.x

[CIT0058] Reiter RJ Tan DX Osuna C Gitto E 2000 Actions of melatonin in the reduction of oxidative stress. A review. Journal of Biomedical Science 7, 444–458. 11060493 10.1007/BF02253360

[CIT0059] Robinson MD McCarthy DJ Smyth GK 2010 edgeR: a Bioconductor package for differential expression analysis of digital gene expression data. Bioinformatics 26, 139–140. 19910308 10.1093/bioinformatics/btp616 PMC2796818

[CIT0060] Selwood T Jaffe EK 2011 Dynamic dissociating homo-oligomers and the control of protein function. Archives of Biochemistry and Biophysics 519, 131–143. 22182754 10.1016/j.abb.2011.11.020 PMC3298769

[CIT0061] Shi H Chan Z 2014 The cysteine2/histidine2-type transcription factor *ZINC FINGER OF ARABIDOPSIS THALIANA 6*-activated *C-REPEAT-BINDING FACTOR* pathway is essential for melatonin-mediated freezing stress resistance in Arabidopsis. Journal of Pineal Research 57, 185–191. 24962049 10.1111/jpi.12155

[CIT0062] Shi H Wang Y Cheng Z Ye T Chan Z 2012 Analysis of natural variation in bermudagrass (*Cynodon dactylon*) reveals physiological responses underlying drought tolerance. PLoS One 7, e53422. 23285294 10.1371/journal.pone.0053422 PMC3532450

[CIT0063] Shi H Wang X Ye T Chen F Deng J Yang P Zhang Y Chan Z 2014a The cysteine2/histidine2-type transcription factor *ZINC FINGER OF ARABIDOPSIS THALIANA 6* modulates biotic and abiotic stress responses by activating salicylic acid-related genes and *C-REPEAT-BINDING FACTOR* genes in Arabidopsis. Plant Physiology 165, 1367–1379. 24834923 10.1104/pp.114.242404 PMC4081343

[CIT0064] Shi H Ye T Chan Z 2013a Comparative proteomic and physiological analyses reveal the protective effect of exogenous polyamines in the bermudagrass (*Cynodon dactylon*) response to salt and drought stresses. Journal of Proteome Research 12, 4951–4964. 23944872 10.1021/pr400479k

[CIT0065] Shi H Ye T Chan Z 2013b Exogenous application of hydrogen sulfide donor sodium hydrosulfide enhanced multiple abiotic stress tolerance in bermudagrass (*Cynodon dactylon* (L). Pers.). Plant Physiology and Biochemistry 71, 226–234. 23974354 10.1016/j.plaphy.2013.07.021

[CIT0066] Shi H Ye T Chan Z 2014b Nitric oxide-activated hydrogen sulfide is essential for cadmium stress response in bermudagrass (*Cynodon dactylon* (L). Pers.). Plant Physiology and Biochemistry 71, 226–234. 24291156 10.1016/j.plaphy.2013.11.001

[CIT0067] Shi H Ye T Chan Z 2014c Comparative proteomic responses of two bermudagrass (*Cynodon dactylon* (L). Pers.) varieties contrasting in drought stress resistance. Plant Physiology and Biochemistry 82, 218–228. 24992888 10.1016/j.plaphy.2014.06.006

[CIT0068] Shi H Ye T Chen F Cheng Z Wang Y Yang P Zhang Y Chan Z 2013c Manipulation of arginase expression modulates abiotic stress tolerance in Arabidopsis: effect on arginine metabolism and ROS accumulation. Journal of Experimental Botany 64, 1367–1379. 23378380 10.1093/jxb/ers400 PMC3598423

[CIT0069] Shi H Ye T Zhong B Liu X Chan Z 2014d Comparative proteomic and metabolomic analyses reveal mechanisms of improved cold stress tolerance in bermudagrass (*Cynodon dactylon* (L). Pers.) by exogenous calcium. Journal of Integrative Plant Biology 71, 226–234. 10.1111/jipb.12167 24428341

[CIT0070] Shi H Ye T Zhong B Liu X Jin R Chan Z 2014e AtHAP5A modulates freezing stress resistance in Arabidopsis through binding to CCAAT motif of *AtXTH21* . New Phytologist 203, 554–567. 24739069 10.1111/nph.12812

[CIT0071] Tal O Haim A Harel O Gerchman Y 2011 Melatonin as an antioxidant and its semi-lunar rhythm in green macroalga *Ulva* sp. Journal of Experimental Botany 62, 1903–1910. 21220782 10.1093/jxb/erq378 PMC3060675

[CIT0072] Tan DX Chen LD Poeggeler B Manchester L Reiter RJ 1993 Melatonin: a potent, endogenous hydroxyl radical scavenger. Endocrine Journal 1, 57–60.

[CIT0073] Tan DX Hardeland R Manchester LC Korkmaz A Ma S Rosales-Corral S Reiter RJ 2012 Functional roles of melatonin in plants, and perspectives in nutritional and agricultural science. Journal of Experimental Botany 63, 577–597. 22016420 10.1093/jxb/err256

[CIT0074] Tan DX Hardeland R Manchester LC Poeggeler B Lopez-Burillo S Mayo JC Sainz RM Reiter RJ 2003 Mechanistic and comparative studies of melatonin and classic antioxidants in terms of their interactions with the ABTS cation radical. Journal of Pineal Research 34, 249–259. 12662346 10.1034/j.1600-079x.2003.00037.x

[CIT0075] Tan DX Manchester LC Helton P Reiter RJ 2007a Phytoremediative capacity of plants enriched with melatonin. Plant Signaling and Behavior 2, 514–516. 19704544 10.4161/psb.2.6.4639 PMC2634354

[CIT0076] Tan DX Manchester LC Reiter RJ Plummer BF 1999 Cyclic 3-hydroxymelatonin: a melatonin metabolite generated as a result of hydroxyl radical scavenging. Biological Signals and Receptors 8, 70–74. 10085465 10.1159/000014571

[CIT0077] Tan DX Manchester LC Terron MP Flores LJ Reiter RJ 2007b One molecule, many derivatives: a never-ending interaction of melatonin with reactive oxygen and nitrogen species? Journal of Pineal Research 42, 28–42. 17198536 10.1111/j.1600-079X.2006.00407.x

[CIT0078] Thimm O Bläsing O Gibon Y Nagel A Meyer S Krüger P Selbig J Müller LA Rhee SY Stitt M 2004 MAPMAN: a user-driven tool to display genomics data sets onto diagrams of metabolic pathways and other biological processes. The Plant Journal 37, 914–939. 14996223 10.1111/j.1365-313x.2004.02016.x

[CIT0079] Venegas C García JA Escames G Ortiz F López A Doerrier C García-Corzo L López LC Reiter RJ Acuña-Castroviejo D 2012 Extrapineal melatonin: analysis of its subcellular distribution and daily fluctuations. Journal of Pineal Research 52, 217–227. 21884551 10.1111/j.1600-079X.2011.00931.x

[CIT0080] Wang L Zhao Y Reiter RJ He C Liu G Lei Q Zuo B Zheng XD Li Q Kong J 2014 Changes in melatonin levels in transgenic ‘Micro-Tom’ tomato overexpressing ovine *AANAT* and ovine *HIOMT* genes. Journal of Pineal Research 56, 134–142. 24138427 10.1111/jpi.12105

[CIT0081] Wang P Sun X Chang C Feng F Liang D Cheng L Ma F 2013a Delay in leaf senescence of *Malus hupehensis* by long-term melatonin application is associated with its regulation of metabolic status and protein degradation. Journal of Pineal Research 55, 424–434. 24103092 10.1111/jpi.12091

[CIT0082] Wang P Sun X Li C Wei Z Liang D Ma F 2013b Long-term exogenous application of melatonin delays drought-induced leaf senescence in apple. Journal of Pineal Research 54, 292–302. 23106234 10.1111/jpi.12017

[CIT0083] Wang P Yin L Liang D Li C Ma F Yue Z 2012 Delayed senescence of apple leaves by exogenous melatonin treatment: toward regulating the ascorbate–glutathione cycle. Journal of Pineal Research 53, 11–20. 21988707 10.1111/j.1600-079X.2011.00966.x

[CIT0084] Weeda S Zhang N Zhao X Ndip G Guo Y Buck GA Fu C Ren S 2014 Arabidopsis transcriptome analysis reveals key roles of melatonin in plant defense systems. PLoS One 9, e93462. 24682084 10.1371/journal.pone.0093462 PMC3969325

[CIT0085] Yang Z Li Z Bickel DR 2013 Empirical Bayes estimation of posterior probabilities of enrichment: a comparative study of five estimators of the local false discovery rate. BMC Bioinformatics 14, 87. 23497228 10.1186/1471-2105-14-87 PMC3658916

[CIT0086] Yin L Wang P Li M 2013 Exogenous melatonin improves Malus resistance to *Marssonina apple blotch* . Journal of Pineal Research 54, 426–434. 23356947 10.1111/jpi.12038

[CIT0087] Zhang N Zhang HJ Zhao B 2014 The RNA-seq approach to discriminate gene expression profiles in response to melatonin on cucumber lateral root formation. Journal of Pineal Research 56, 39–50. 24102657 10.1111/jpi.12095

[CIT0088] Zhang N Zhao B Zhang HJ Weeda S Yang C Yang ZC Ren S Guo YD 2013 Melatonin promotes water-stress tolerance, lateral root formation, and seed germination in cucumber (*Cucumis sativus* L.). Journal of Pineal Research 54, 15–23. 22747917 10.1111/j.1600-079X.2012.01015.x

[CIT0089] Zhao Y Qi LW Wang WM Saxena PK Liu CZ 2011 Melatonin improves the survival of cryopreserved callus of *Rhodiola crenulata* . Journal of Pineal Research 50, 83–88. 21073518 10.1111/j.1600-079X.2010.00817.x

[CIT0090] Zhao Y Tan DX Lei Q Chen H Wang L Li QT Gao Y Kong J 2013 Melatonin and its potential biological functions in the fruits of sweet cherry. Journal of Pineal Research 55, 79–88. 23480341 10.1111/jpi.12044

