# Supplementary material for: Comparative physiological, metabolomic, and transcriptomic analyses reveal mechanisms of improved abiotic stress resistance in bermudagrass [Cynodon dactylon (L). Pers.] by exogenous melatonin
Source: J Exp Bot. 2014 Sep 15;66(3):681–94. doi: 10.1093/jxb/eru373 (PMC4321537; doi:10.1093/jxb/eru373)
Supplement: Supplementary Data [file supp_66_3_681__index.html]

Comparative physiological, metabolomic, and transcriptomic analyses reveal mechanisms of improved abiotic stress resistance in bermudagrass [Cynodon dactylon (L). Pers.] by exogenous melatonin — Comparative physiological, metabolomic, and transcriptomic analyses reveal mechanisms of improved abiotic stress resistance in bermudagrass [Cynodon dactylon (L). Pers.] by exogenous melatonin — Supplementary Data 

# Comparative physiological, metabolomic, and transcriptomic analyses reveal mechanisms of improved abiotic stress resistance in bermudagrass [*Cynodon dactylon* (L). Pers.] by exogenous melatonin

## Supplementary Data

Data files

**Files in this Data Supplement:**

- Supplementary Data - Supplementary Data
- Supplementary Data - Supplementary Data
