# Supplementary material for: Comparative physiological, metabolomic, and transcriptomic analyses reveal mechanisms of improved abiotic stress resistance in bermudagrass [Cynodon dactylon (L). Pers.] by exogenous melatonin
Source: J Exp Bot. 2014 Sep 15;66(3):681–94. doi: 10.1093/jxb/eru373 (PMC4321537; doi:10.1093/jxb/eru373)
Supplement: Supplementary Data [file supp_eru373_jexbot132589_file002.pdf]

Comparative physiological, metabolomic and transcriptomic analyses reveal mechanisms of improved abiotic stress resistance in bermudagrass (*Cynodon dactylon* (L). Pers.) by exogenous melatonin

Haitao Shi, Chuan Jiang, Tiantian Ye, Dunxian Tan, Russel J Reiter, Heng Zhang, Renyi Liu, and Zhulong Chan

SUPPLEMENTARY DATA

Table S1. The specific primers used for real-time PCR.

Table S2. Concentrations of 54 metabolites in 28-day-old bermudagrass plants after different treatments (control or 20 µM melatonin) under control, 300 mM NaCl, drought, and cold (4°C) stress conditions for 14 days. The concentrations of metabolites are expressed as µg•g^{-1} FW. Three biological replicates were used for each group.

Table S3. Summary of RNA-Seq data.

Table S4. List of up-regulated genes in melatonin-treated bermudagrass plants.

Table S5. List of down-regulated genes in melatonin-treated bermudagrass plants.

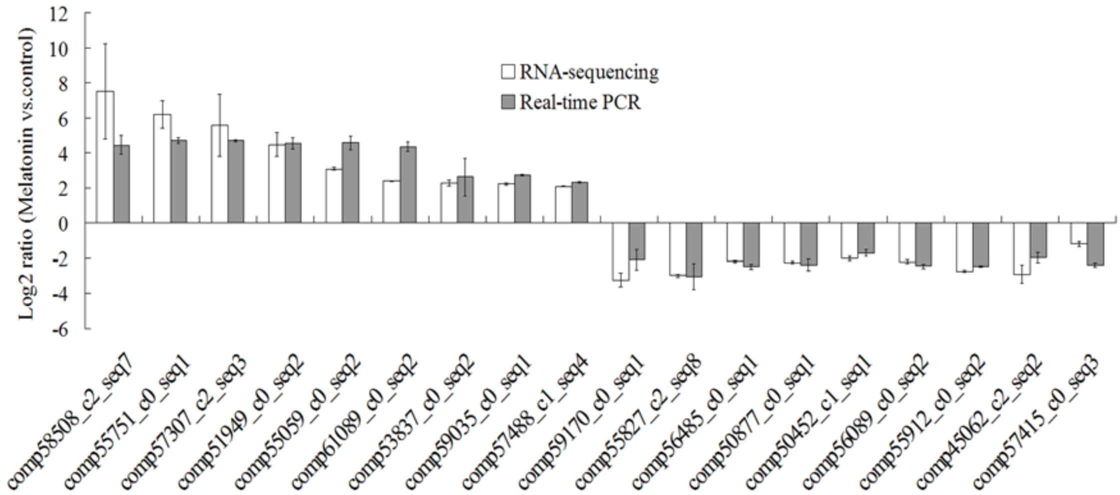

Figure S1. Validation of differentially expressed genes by quantitative real-time PCR.

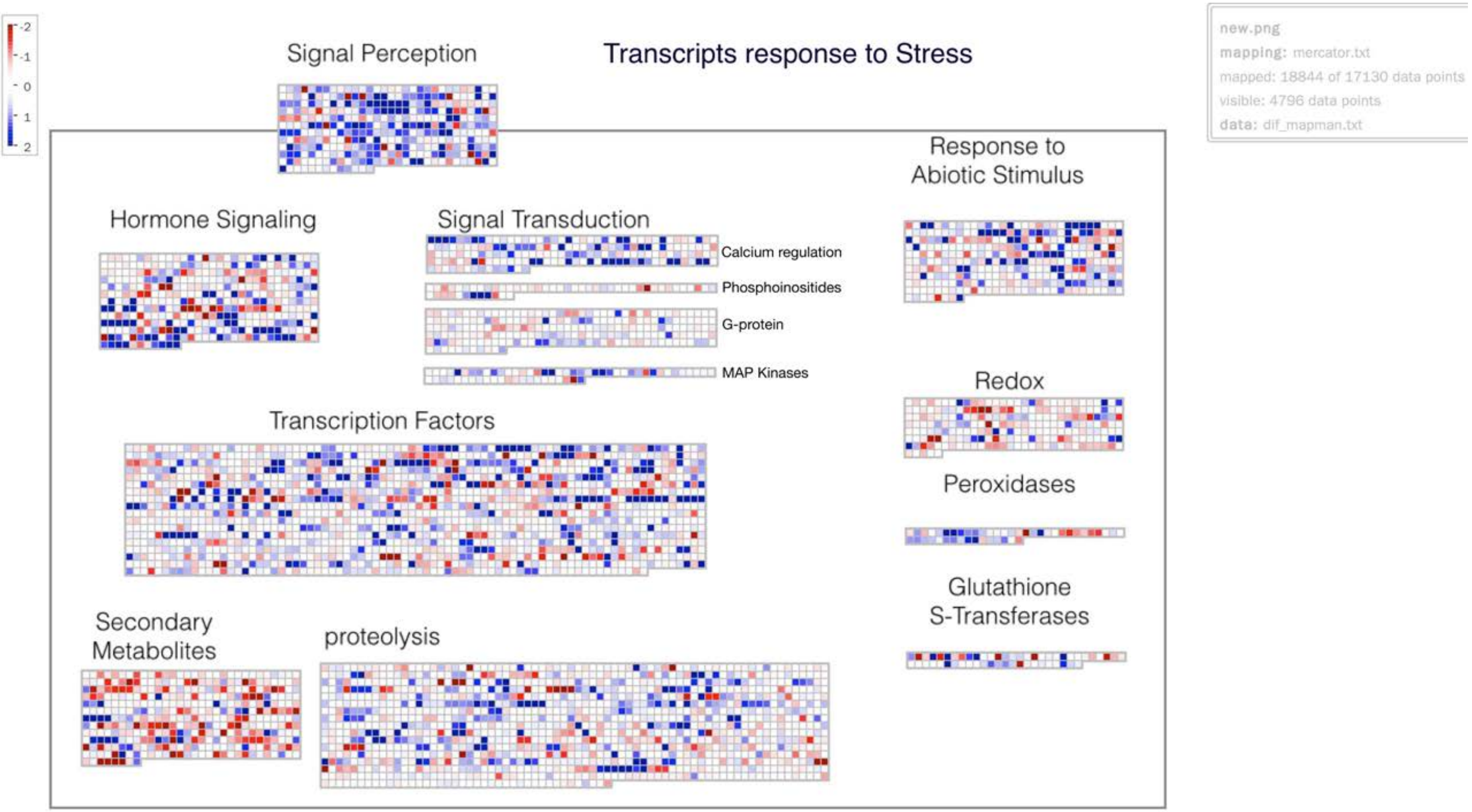

Figure S2. Effect of exogenous melatonin treatment on stress-related pathways in bermudagrass. Transcripts participate in the same pathway or homologs were represented by a set of closely-connected cubes, and the 2 based logarithm of fold change value (log_2FC) were denoted by colors.
